# Influence of Sealing Surface Microstructure Characteristics on Flow Resistance and Leakage Between Contact Surfaces

**DOI:** 10.3390/ma18194474

**Published:** 2025-09-25

**Authors:** Przemysław Jaszak, Anna Piwowar, Marcin Bieganowski

**Affiliations:** Faculty of Mechanical and Power Engineering, Wroclaw University of Science and Technology, Wybrzeze Wyspianskiego 27, 50-370 Wroclaw, Poland; marcin.bieganowski@pwr.edu.pl

**Keywords:** flange-bolted joint, gasket, tightness improvement, CFD

## Abstract

This paper presents the results of preliminary numerical and experimental studies concerning the sealing performance of static seals (gaskets) with geometrically designed sealing surface microstructures. The concept of the microstructure, inspired by the operating principle of Tesla’s one-way valve, relies on the generation of localized flow circulation within the microchannels formed between the contact surfaces, which increases flow resistance and reduces leakage. CFD simulations were performed to assess the influence of the geometric parameters of the microstructure on the leakage rate. The numerical calculations demonstrated that introducing microstructures into the gap formed between the contact interfaces can significantly reduce leakage, with the most critical geometric parameters being the gap width between the microprotrusions, their packing density, and their height. Experimental studies confirmed the higher sealing performance of structured gaskets compared to quasi-smooth gaskets, particularly at lower contact pressures. An analysis of the effective contact surface revealed that the improvement in tightness is a result of both the local intensification of the contact pressure and the flow effects induced by the microprotrusions. The results obtained confirm that an appropriately designed surface microstructure can substantially enhance the sealing performance of flange-bolted joints, even under relatively low clamping loads.

## 1. Introduction

Gaskets are widely used in most machines, devices, and piping components. Their function is to provide protection against the leakage of aggressive substances into the environment, as well as to protect the interior from the harmful effects of environmental factors (such as salt water). They constitute critical components that have a direct impact on the operational safety of machines, devices, and piping components, as well as on the level of environmental pollution and the energy efficiency of the installations/processes, resulting from the minimization of mass losses. When analyzing the operating conditions of a gasket in a joint, it should be noted that the contact surfaces are always characterized by a certain roughness and waviness, and thus their conformity is achieved only over a limited portion of the nominal contact area. Consequently, microgaps appear between the surfaces, through which leakage of the sealed medium may occur. The level of joint tightness obtained depends primarily on the microstructure parameters of the sealing and mating surfaces, such as roughness, mechanical properties of the materials used (stiffness, porosity, or resistance to aging), and macroscopic contact conditions, including joint clamping, pressure of the sealed medium, and the resulting distributions of contact pressure. The influence of the factors mentioned above on joint tightness has been described, among others, in [1,2,3].

The influence of surface roughness on fluid permeability at contact interfaces has been extensively analyzed in a wide range of publications. Predictive models of leakage through rough surfaces have been derived. Persson et al. [4] developed models for contact and leakage estimation through rubber seals [5,6,7] and metal gaskets [8]. Zhang et al. [9,10] developed a fluid leakage model for the interface of metal sealing surfaces, based on the Morag–Etsion fractal contact model [11] and the fractal theory of fluid transport in porous media introduced by Yu et al. [12,13]. Pérez-Ràfols et al. [14] described a leakage model for metal gaskets with contact surface structures manufactured by turning, in which both the spiral surface microstructure and its roughness were considered. Jaszak et al. [15] modeled fluid permeability at the interface between flat, parallel plates and sharp-edged ridges of a metal gasket by applying the finite element method (FEM), Darcy’s equation, and fractal theory.

For modeling leakage for various types of seals, including shaft radial seals [16] and labyrinth seals [17], computational fluid dynamics (CFD) is also applied. Zhang et al. [18] proposed a solution that integrates FEM and CFD for modeling leakage at the interface between two stationary, rough sealing surfaces.

Other researchers focused on analyzing the influence of gasket design on the distribution of contact pressure and the tightness of the joint, employing FEM and experimental studies. Haruyama et al. [19,20,21] developed and optimized a corrugated metal gasket, utilizing the material’s elasticity to improve sealing performance. Du et al. [22] carried out an optimization of a metal gasket with respect to the distribution of contact pressure on its surface. Zhou et al. [23] performed an elastoplastic contact mechanics analysis, analytically determining the contact width and the distribution of contact pressure for a lenticular metal gasket, based on Hertz’s contact theory. The analytical results were verified using FEM. The data obtained were applied to evaluate the influence of the contact diameter, the gasket inclination angle, and the joint operating conditions on the leakage rate. Adamek et al. [24] described a design method for spiral wound gaskets with PTFE filling and developed a gasket with an asymmetric profile of windings, which exhibited higher stiffness and tightness compared to conventional spiral wound gaskets with a symmetric profile of windings.

The total leakage from a gasket in a bolted flange joint should be considered in the context of two components. One of them is the leakage component that occurs at the interface of the joined surfaces (interfacial leak), while the other component is the leakage through the porous structure of the gasket material (permeation leak). In [25], a study was carried out to determine what fraction of the total leakage is constituted by leakage through the gasket. Based on the results of the available studies, it can be stated that a complete elimination of leakage is practically impossible; however, the assumption of achieving so-called zero-emission has motivated designers to develop new sealing solutions. Improvement of the tightness can be considered in two ways: by applying materials characterized by low permeability and, on the other hand, soft enough to fill all surface irregularities at relatively low loads. This is achieved, among others, by the use of metal or semi-metal gaskets, which allow for a significant reduction in leakage through the gasket because of the presence of a metal core. The results of such work have been presented, among others, in [26,27]. Another approach to minimizing leakage is the reduction in the interfacial leakage component. This is accomplished through the local intensification of contact pressure. A serrated design of the metal core [28] is one such construction. Another method is the geometrical structuring of the sealing surface, aimed at increasing the flow resistance of the sealed medium. An analysis of such a solution was presented in [29]. The authors reported the results of a study on a rotary shaft seal, in which a clearance seal with a structured sealing surface of the honeycomb type was applied. Example solutions for static seals with a regular sealing surface structure were presented in [30]. According to the patent description of this solution, a regular sealing surface structure can reduce leakage by several orders of magnitude compared to a smooth gasket.

The application of geometrical structuring of the sealing surface has so far been analyzed primarily in the context of friction reduction in dynamic joint seals, as described in [31,32,33]. There is a knowledge gap concerning the influence of the geometrical structuring of the sealing surfaces in gaskets on the tightness of the joint. This paper presents an innovative approach to the design of the external sealing surface, inspired by the operating principle of Tesla’s one-way valve, in which flow is undisturbed in the forward direction, while in the reverse direction, it is strongly restricted due to the asymmetrical channel geometry that induces vortex structures and significantly increases flow resistance [34,35]. The proposed solution involves the formation of a series of microprotrusions on the sealing surface whose function is to induce local circulation of the sealed medium. It is assumed that this phenomenon may lead to an increase in flow resistance at the interface between the sealing and mating surfaces, and thus to an improvement in joint tightness. The scientific contribution of this work is the demonstration that sealing surface microstructuring, designed to increase flow resistance, can effectively reduce leakage in flange-bolted joints, along with an evaluation of the influence of selected microstructure parameters on sealing performance.

The main objective of the study was to evaluate the influence of characteristic dimensions that describe the microstructure of the external sealing surface on the leakage rate from the joint. Preliminary analyses were performed using CFD numerical simulations and experimental studies.

## 2. Materials and Methods

### 2.1. Research Object

The research objects were gaskets with inner and outer diameters compliant with the standard [36], and a thickness of 3 mm, used in the flanges designated PN 40 DN 40 according to the standard [37]. The analysis covered gaskets with a geometrically modified microstructure of the sealing surfaces and, for comparison purposes, the reference gaskets G0 with unmodified, quasi-smooth sealing surfaces.

The surface modification was carried out based on the following assumptions:

- The depth of the microstructure, perpendicular to the formed surface (the height of the microprotrusions), equals d;
- The microstructure is located within a closed, concentric limiting ring positioned at the outer diameter of the gasket;
- The microstructure consists of a series of concentrically arranged microprotrusions forming a staggered pattern in the radial direction;
- The microprotrusions are formed as split ellipses, with the minor axis oriented parallel to the radial direction and the split located on the side of the sealed medium. However, in one of the variants tested, unsplit ellipses were applied;
- The microprotrusions were arranged on pitch diameters at constant intervals, with equal spacing between the outer walls in both the radial and circumferential directions. The number of microprotrusions on each pitch diameter is $n_{circ}$, while the number of rows in the radial direction is $n_{rad}$;
- On every second pitch diameter, the pattern was angularly shifted by an angle equal to $360{}^{\circ}/(2n_{circ})$;
- All ellipses are characterized by the same length of the minor axis.

**Figure 1:**
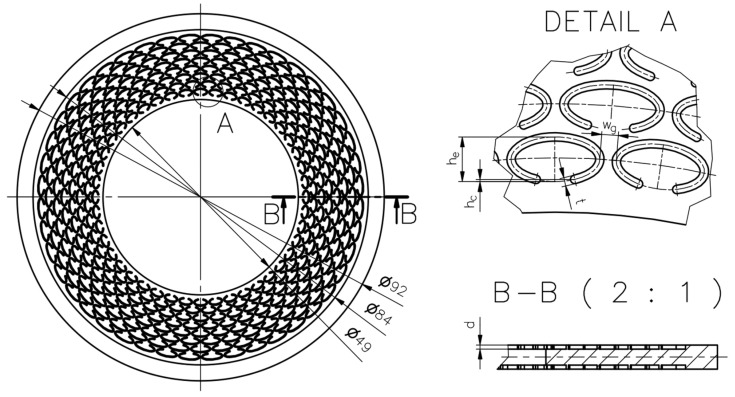
Microstructure geometry of the external sealing surfaces.

An example of the geometry of the microstructure is shown in Figure 1. Table 1 contains the characteristic dimensions of the microstructure of all gasket variants tested.

The gasket variants were designed to evaluate the influence of selected parameters of the microstructure of the external sealing surface on the leakage rate through the gasket. Variant G1 represents the initial design among the structured gaskets with elliptical microprotrusions—its geometric parameters were adopted as the starting point for further modifications. Variant G2 was designed to evaluate the influence of changing the gap width between the microprotrusions $w_{g}$. Variant G3 allows for a preliminary analysis of the effect of changing the height of the split of the ellipse $h_{c}$. Variant G4 was designed to assess the influence of the height of the microprotrusions d. Variant G5 enables a preliminary analysis of the influence of the thickness of the microprotrusions t while maintaining a constant gap width between their walls. Variant G6 is intended to evaluate the influence of the packing density of the microprotrusions (by changing the number of $n_{circ}$ and $n_{rad}$), under the assumption of proportional changes in their main dimensions ($h_{e}$, $h_{c}$).

### 2.2. Methodology of Numerical Calculations

To estimate the influence of the geometric parameters of the microstructure of the external sealing surface on the generated flow resistance (leakage rate), CFD was employed. For the preliminary numerical calculations, simplified flow gap geometries corresponding to the gaskets described in Section 2.1 were used. The computational model assumed certain simplifications of geometry compared to the actual surface of the analyzed variants, namely:

- The use of a periodic segment of the upper/lower gap, omitting the ring section at the outlet—to evaluate the influence of the microgeometry on leakage;
- No deformation of the protrusions during gasket operation—constant gap height;
- The sealed medium was water;
- The leak was modeled considering only the flow at the interface between the sealing surfaces, whereas in reality it also includes flow through the porous structure of the material;
- The roughness and waviness of the flanges and the roughness of the microstructure walls were not included—only the effect of the microprotrusion geometry on leakage was assessed.

In the CFD calculations, the reference Variant G0* was assumed as a ring gap with a height of 0.1 mm.

**Figure 2:**
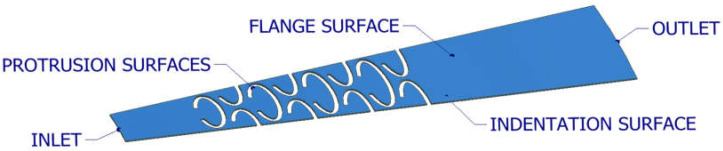
CFD fluid body.

The fluid bodies obtained represented a fraction of the total gap volume, which had a critical impact on the reduction in the mesh size. An example of a fluid body is shown in Figure 2.

The numerical calculations were carried out using Ansys CFX 2024 R2. The simulations were performed in two stages: first in steady-state mode, obtaining preliminary velocity and pressure distributions, which were then used as initial conditions for the transient calculations.

The Shear Stress Transport (SST) with the Gamma Theta transition turbulence model was applied due to the nature of the flow in the narrow gaps investigated, where near-wall effects play a dominant role. By combining the advantages of the classical k-ω and k-ε models, the SST model ensures computational stability in the core region of the flow and provides an accurate representation of the near-wall layers, being insensitive to mesh resolution, thereby enabling reliable prediction of flows with strong adverse pressure gradients and separation [38,39].

In addition, the SST model allows the incorporation of the Gamma Theta transition model, which describes the laminar-turbulent transition phenomenon. This extension is particularly useful for flows in narrow gaps, where transitional effects are expected, and it is recommended in the Ansys documentation as a general-purpose model [40]. The application and validation of the Gamma Theta transition model in combination with the SST turbulence model have been reported, among others, in [41,42,43].

Already at the stage of the discretization of the flow volume, it was recognized that, for some analyzed cases, the flow might fall within the transitional regime. Preliminary estimates indicated that the minimum Reynolds number could reach values of approximately Re ~ 3000, which for certain variants would place the flow in the transitional range. This justified the inclusion of the Gamma Theta transition model as part of the adopted turbulence modeling approach.

In all cases analyzed, the working fluid was water at a temperature of 20 °C. At the inlet and outlet, boundary conditions were assumed in the form of absolute pressures: 41 bar and 1 bar, respectively. A time step of 1 · 10^−6^ s was adopted to maintain the Courant-Friedrichs-Lewy (CFL) number ~ 1. The total simulation time was set to 1 · 10^−3^ s. The convergence criterion was defined as the root-mean-square (RMS) residuals dropping below 10^−5^ at each time step.

The discretization of the flow volume was carried out in such a way as to capture the flow phenomena that occur in the gap. For this reason, the mesh size was primarily influenced by the value of the y+ parameter, which, according to the requirements of the turbulence model, should satisfy y+ < 2 [44], and the “aspect ratio” parameter. To create flow volumes, a structured hexahedral mesh with inflation layers was applied. The mesh sizes obtained and their characteristic parameters for the segments are presented in Table 2.

A grid independence test (GIT) was also carried out, which indicated that the optimal number of mesh elements was approximately 20 million. However, due to the requirement of maintaining y+ ~ 1 during transient calculations, finer (oversized) meshes were applied.

During the calculations, the value of the mass flow rate was monitored. The average leakage in a single simulation was defined as the mean value of the mass flow rate determined over the second half of the simulation time (5 · 10^−4^–1 · 10^−3^ s), when the monitored flow values exhibited negligible temporal variations and did not show any significant trend of further variation. Based on the recorded values, a flow reduction factor (FRF) was determined after the introduction of the microprotrusions into the flow gap, defined as the ratio of the mass flow rate for the reference geometry (G0*) to that of the geometry variant with the microprotrusions $q_{m,n}$:

(1) $$FRF\;=\;\frac{q_{m,0}}{q_{m,n}}.$$

The factors obtained served as the basis for selecting the geometries for testing on the experimental test stand under real operating conditions.

### 2.3. Methodology of Experimental Studies

To verify the influence of the geometry (formation) of the microstructure of the sealing surface on the tightness of the flange-bolted joint, experimental tests were performed. The gasket variants described in Section 2.1 were manufactured from Formlabs Black Resin V5 photopolymer resin (Formlabs, Somerville, MA, USA) using stereolithography (SLA) 3D printing on a Formlabs Form 4 printer (Formlabs, Somerville, MA, USA). The microstructure was an integral part of the CAD model of each gasket variant and was directly reproduced during the printing process without any additional post-processing. The prints were cured at room temperature for 7 min under UV exposure in a Formlabs Form Cure device (Formlabs, Somerville, MA, USA). The mechanical properties of the resin after curing are given in Table 3.

**Figure 3:**
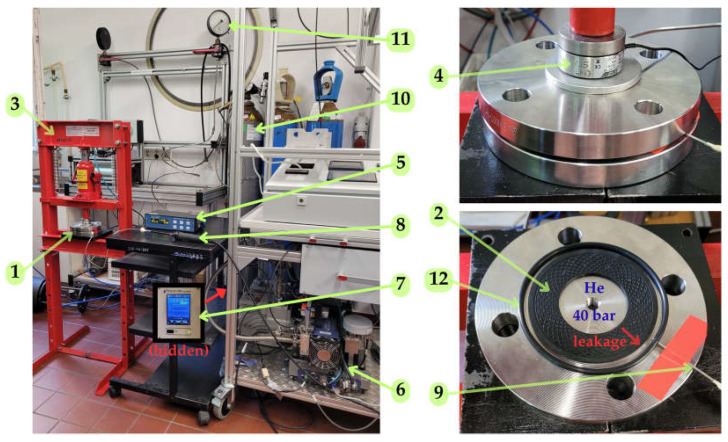
Test stand; 1—flange joint, 2—test sample, 3—hydraulic press, 4—force sensor, 5—force monitor, 6—helium leak detector, 7—leakage monitor, 8—sniffer, 9—sniffer connector, 10—helium canister, 11—helium pressure gauge, 12—secondary seal.

The experimental investigation was carried out on a dedicated test stand, shown in Figure 3.

The experimental procedure consisted of properly clamping the tested gasket placed between two flat metal flanges, introducing helium at a constant pressure into the interior of the sealed flanges, and subsequently measuring the leakage. The flange joint was clamped using a hydraulic press with a maximum compressive force of 12 tons. The clamping force was measured with a strain gauge sensor type LC20-18 ZEPWN (ZEPWN, Marki, Poland). The total helium leakage was collected in a collector chamber (formed between the tested gasket and a rubber O-ring), from which it was then drawn in through a sniffer probe and analyzed by a spectrometric helium leak detector. The specification of the measuring instruments used is given in Table 4.

The gaskets were tested under clamping loads of the flange joint in the range of 60 to 110 kN, increased in increments of 10 kN. The applied helium gauge pressure was 40 bar. The tests were carried out at an ambient temperature of approximately 20 °C. At each measurement point, after a stabilization time of 20 min, the volumetric leakage rate q_v_ was recorded, calculated as the average of five consecutive readings. Each gasket variant was investigated in two independent tests, and the mean value of both measurements was used for the analysis of the results. Images of selected samples before and after testing are shown in Table 5.

The measured volumetric leakage rate $q_{v}$ was converted to the mass leakage rate $q_{m}$ using the ideal gas equation [46]:

(2) $$p\cdot V\;=\frac{m}{M}\cdot R\cdot T,$$

where p is the gas pressure in Pa, V is the gas volume in m^3^, m is the gas mass in g, M is the molar mass of the gas in g/mol, R is the gas constant in J/(mol·K), and T is the gas temperature in K.

After transforming Equation (2), a relation was obtained that allows the determination of the mass leakage rate $q_{m}$ in g/s:

(3) $$q_{m}\;=\frac{q_{v}\cdot M}{R\cdot T},$$

where $q_{v}$ is the volumetric leakage rate measured by the leak detector, expressed in units of mbar·L/s.

The mass leakage rate of the sealed medium was normalized to the average gasket circumference and graphically presented as a function of the contact pressure (referred to the gasket surface), according to the approach applied in the standard [47]. In the first approach, it was assumed that the contact pressure for the structured gaskets refers to the nominal surface, corresponding to the area of a smooth gasket. In the second approach, it was assumed that the pressure was referred to the effective contact surface of the gasket with the flange (the area of the microprotrusions). The effective surfaces area was determined directly from the CAD models as the sum of the frontal surfaces of the microprotrusions, representing the functional contact surfaces. The force applied to the gasket surface (residual clamping force) was determined as the difference between the compressive force applied to the flange joint (the force obtained from the sensor) and the hydrostatic force resulting from the pressure of the sealed medium.

## 3. Results and Discussion

### 3.1. Numerical Calculations

**Figure 4:**
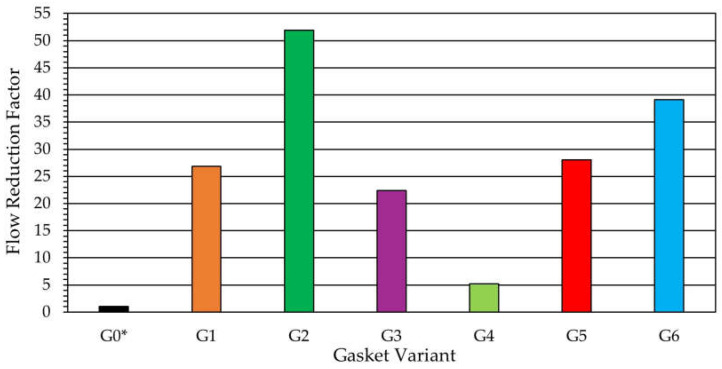
Flow reduction factor across gasket variants.

Figure 4 presents the quantitative results of the CFD simulations, i.e., the values of the flow reduction factor for the investigated geometries. From the bar chart of the FRF values for the analyzed microstructure variants, it follows that the geometric parameters of the microstructure have a significant influence on the level of leakage reduction. The analysis of the results obtained demonstrated that the introduction of the microprotrusions into the flow gap causes a substantial reduction in leakage, ranging from about five up to as much as fifty-two times compared to the reference variant. Among the structured variants, the highest level of leakage reduction was obtained for Variant G2 (FRF ~ 52), in which the gap width between the microprotrusions $w_{g}$ was reduced. The high effectiveness was also demonstrated by Variant G6 (FRF ~ 39), in which the packing density of the microprotrusions was decreased (by changing the numbers $n_{circ}$ and $n_{rad}$). Comparable FRF values (~22–28) were obtained for Variant G1 (the initial design among the structured gaskets, from which further modifications were made), Variant G3 (the variant with reduced height of the split of the ellipse $h_{c}$) and Variant G5 (the variant with reduced thickness of the microprotrusions t). The lowest effectiveness, which provided only about a five-fold reduction in leakage, was obtained for Variant G4, in which the height of the microprotrusions d was increased. The results suggest that the geometric parameters with the greatest influence on the effectiveness of the microgeometry are the gap width between the microprotrusions $w_{g}$, the packing density of the microprotrusions ($n_{circ}$, $n_{rad}$) and their height d. The height of the split of the ellipse $h_{c}$ and the thickness of the microprotrusions t did not have a significant influence on the flow resistance through the gap.

For the reference variant (G0*) and Variant G2, which exhibited the lowest leakage, an additional analysis of the pressure and velocity distributions was performed in the mid-plane cross-section of the ring gap, corresponding to the symmetry plane of the geometric configuration.

**Figure 5:**
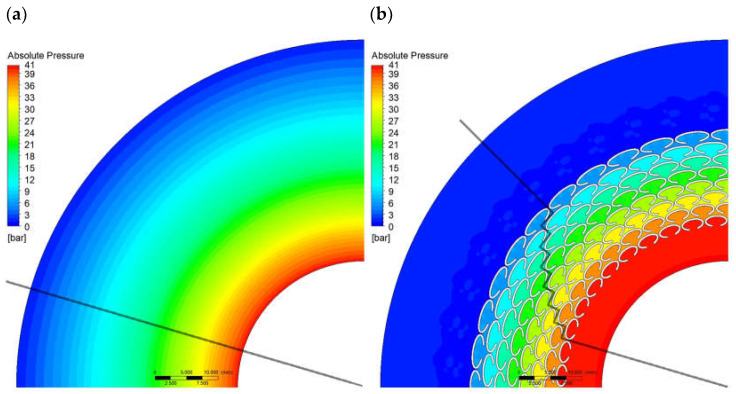
Absolute pressure distribution; (**a**) for G0*, (**b**) for G2.

**Figure 6:**
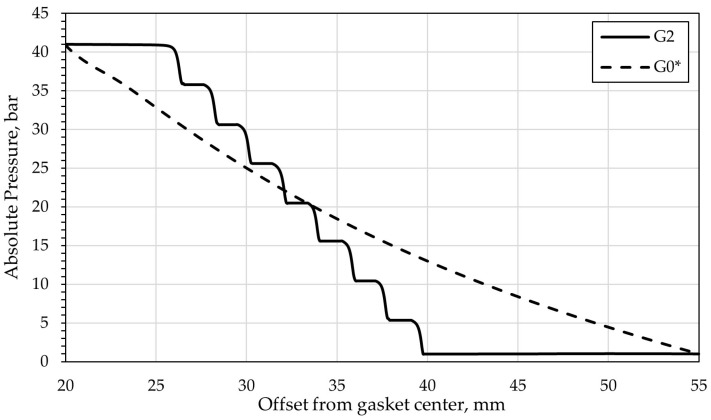
Absolute pressure as a function of offset from the gasket center; (dashed line) for G0*, (full line) for G2.

Figure 5 compares the absolute pressure distributions for Variants G0* and G2. In the visualizations, lines are indicated along which the pressure values were determined as a function of the distance from the gasket center, and these are presented in Figure 6. In the case of the microstructured variant, due to rotational symmetry, the cross-section from which the pressure values were obtained was rotated so as to pass through the centers of the gaps between the ellipses. The comparison of the characteristics obtained for G0* and G2 presents the change in the pressure drop profile in the flow gap resulting from the introduction of the microprotrusions. For Variant G0*, a smooth, uniform pressure decrease from the inlet to the outlet of the fluid is observed, whereas for Variant G2 a characteristic steep pressure drop can be seen between successive rows of ellipses, amounting to approximately 5 bar per segment. The analysis of both characteristics indicates that the gasket with microgeometry exhibits greater throttling than the reference solution, at a radius of 40 mm, Variant G2 reaches ambient pressure, while G0* remains at about 13 bar. This shows a significant increase in flow resistance.

**Figure 7:**
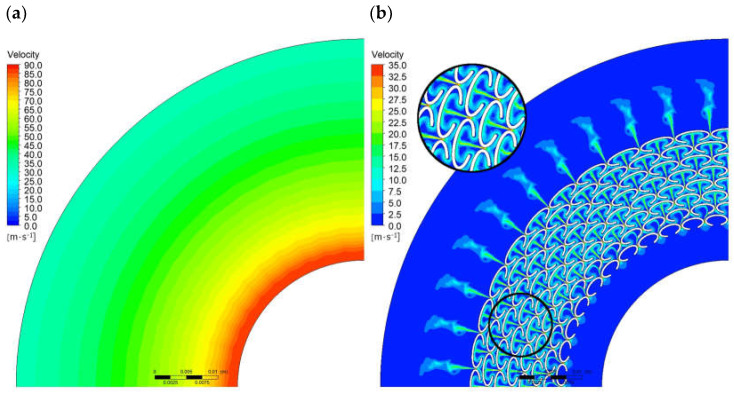
Velocity magnitude distribution; (**a**) for G0*, (**b**) for G2.

**Figure 8:**
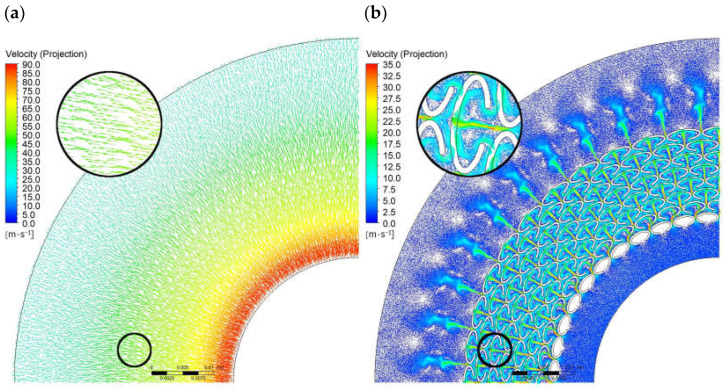
Velocity vectors; (**a**) for G0*, (**b**) for G2.

Figure 7 and Figure 8 present a comparison of the velocity magnitude distributions and the velocity vectors for Variants G0* and G2. Analyzing the flow patterns, it can be observed that the leakage through Variant G0* is strictly radial, with the fluid velocity changing uniformly along the radius. In the case of Variant G2, the velocity distributions are non-uniform, with local changes in the flow direction observed in the regions of the ellipses, forming circulation zones that result in increased flow resistance and reduced fluid velocity. The maximum fluid velocity for Variant G0* reaches approximately 87 m/s, while for Variant G2 it is about 33 m/s.

### 3.2. Experimental Studies

**Figure 9:**
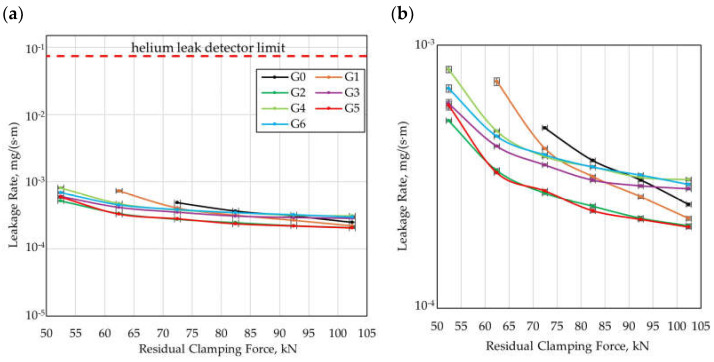
Leakage rate as a function of residual clamping force; (**a**) wide range of leakage rate, (**b**) detailed view.

**Figure 10:**
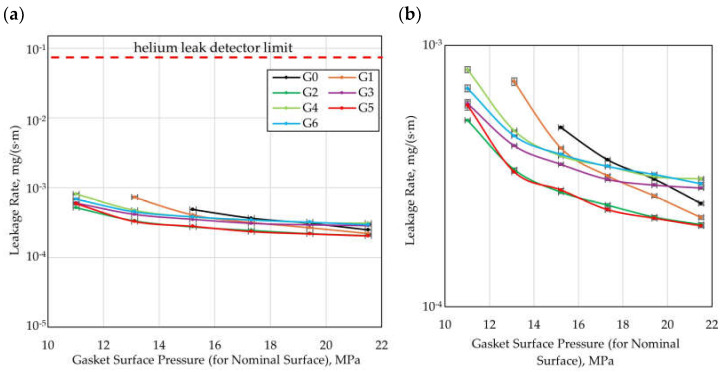
Leakage rate as a function of gasket surface pressure (for nominal surface); (**a**) wide range of leakage rate, (**b**) detailed view.

Within the experimental investigation, leakage characteristics were determined for all gasket variants. The results were presented as a function of the gasket load. Figure 9 presents the leakage rate as a function of the residual clamping force applied to the gasket surface, while Figure 10 presents the leakage characteristics as a function of the contact pressure of the gasket referred to the nominal surface (corresponding to the area of a smooth gasket). The residual clamping force was calculated as the difference between the compressive force applied to the flange joint and the hydrostatic force resulting from the pressure of the sealed medium. It should be noted that for the residual clamping force values corresponding to the lower measurement range (52.5–62.5 kN, generating a contact pressure of 11.0–13.1 MPa), in the case of two gasket variants, i.e., G0 and G1, no measurement points were recorded on the plot. This was the result of exceeding the maximum leakage rate threshold detectable by the helium detector.

From the analysis of the plots in Figure 9 and Figure 10, it follows that all gaskets tested with a structured surface, i.e., G1–G6, exhibited higher tightness than the smooth gasket (G0) within the residual clamping force range of 52.5–82.5 kN. All gasket variants with microstructure (G1–G6) reached the tightness class of 1 · 10^−3^ at a residual clamping force in the range of 52.5–62.5 kN, while the reference gasket (G0) reached this tightness class at a clamping force of 72.5 kN.

In the upper measurement range (above 92.5 kN), higher tightness than the reference gasket (G0) was exhibited by Gasket G1 (the initial variant), G2 (the variant with reduced gap width between the microprotrusions $w_{g}$) and G5 (the variant with reduced thickness of the microprotrusions t). In contrast, Gasket G3 (the variant with reduced height of the split of the ellipse $h_{c}$), G4 (the variant with increased height of the microprotrusions d), and G6 (the variant with reduced packing density of the microprotrusions, i.e., lower values of $n_{circ}$ and $n_{rad}$) exhibited lower tightness than the reference variant G0.

The differences in the sealing performance achieved between the individual variants are particularly pronounced at lower values of the residual clamping force, suggesting that the influence of the gasket geometry is most significant in the lower load range. This is most likely due to the fact that at lower clamping levels, the structuring of the external sealing surface induces local circulation of the fluid, leading to partial backflow relative to the direction of the sealed medium and the generation of additional flow resistance. At higher contact pressures, this effect becomes weakened or disappears, most likely as a result of the microstructure deformation.

**Figure 11:**
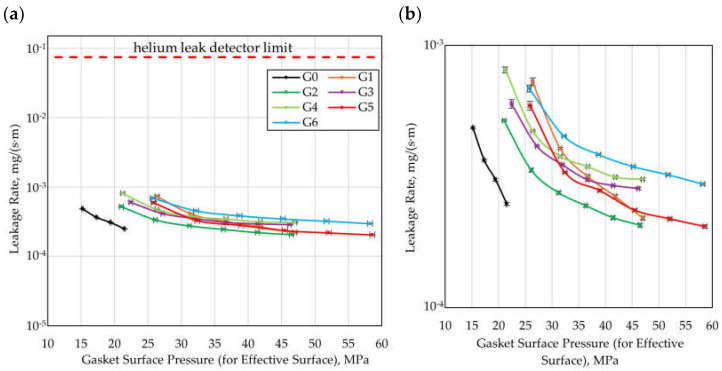
Leakage rate as a function of gasket surface pressure (for effective surface); (**a**) wide range of leakage rate, (**b**) detailed view.

When evaluating the effectiveness of gaskets in terms of the flow resistance generated by the microprotrusions, it is also necessary to account for the influence of the structuring on the contact pressures normalized to the effective contact surface (the area of the microprotrusions). To obtain a more reliable comparison of the variants investigated, Figure 11 presents the leakage rate as a function of the contact pressure referred to the effective contact surface of the gasket with the flange.

Considering the contact pressures referred to the effective contact surface, it can be stated that in the case of structured gaskets (G1–G6), significantly higher contact pressure values are achieved than for the reference gasket (G0), which indicates that the improvement in sealing performance results partly from intensified local contact pressures. Concurrently, the influence of the geometric parameters of the microstructure of the sealing surface on the sealing performance is still evident, even when the results are normalized to the contact pressures for the effective surface.

Among structured gaskets, the highest sealing performance at the lowest contact pressures was achieved by Variant G2. Its effectiveness is consistent with the predictions of the CFD calculations, according to which a reduction in the gap width between the microprotrusions ($w_{g}$) leads to an increase in sealing performance. According to the CFD analysis, an improvement in sealing performance compared to Variant G1 was also expected for Variant G6, resulting from a reduction in the packing density of the microprotrusions (lower values of $n_{circ}$ and $n_{rad}$). However, in the experiment, this effect was only noticeable at the lowest value of the residual clamping force (52.5 kN, corresponding to 21.2 MPa of contact pressure referring to the effective contact surface for G1 and 25.7 MPa for G6). In turn, for Variant G4 (with increased height of the microprotrusions d), the CFD calculations indicated a reduction in sealing performance compared to G1. The experimental results confirmed this effect at higher values of residual clamping force (above 82.5 kN, i.e., 36.7 MPa of contact pressure referred to the effective contact surface for both G1 and G4).

When analyzing leakage as a function of contact pressure, it should be noted that due to the waviness and roughness of the contact surfaces, the actual local contact pressures are not identical to the pressures determined for the effective surface defined in the article. This approach is consistent with the approaches adopted in standards for the evaluation of gasket parameters (e.g., [47]), where the idealization of contact surfaces is assumed.

**Figure 12:**
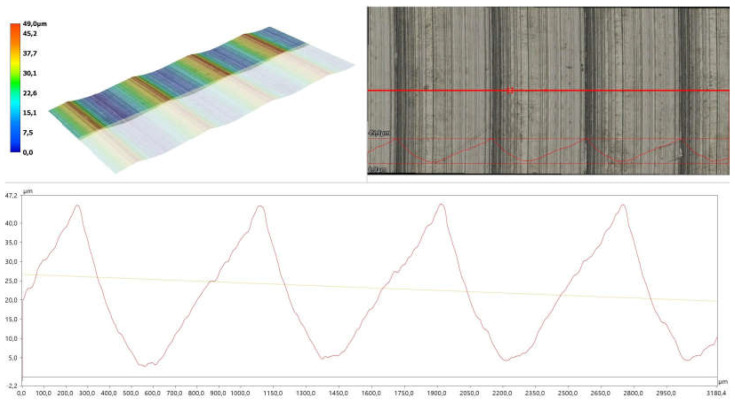
Image and profile of a fragment of the flange surface.

The study was extended to include the measurement of the roughness of the functional surfaces of the gaskets and flanges. The measurements were performed using a KEYENCE VHX-X1F digital microscope (KEYENCE INTERNATIONAL, Mechelen, Belgium) in accordance with the standard [48]. For the surfaces of the printed samples, the values obtained were $R_{a}$ = 1.1 μm and $R_{z}$ = 6.6 μm, while for the flange surfaces they were $R_{a}$ = 10.1 μm and $R_{z}$ = 41.3 μm. Figure 12 presents the image and profile of a fragment of the flange surface.

When analyzing the experimental results and comparing them with the numerical calculations, the following aspects should be noted:

- The numerical calculations were carried out for an undeformed flow gap, while during the experiments the gaskets underwent deformations, as confirmed by the images of the samples before and after testing, presented in Table 5 (concentric contact traces originating from the flange surface are visible, particularly in the ring region of the structured gaskets). The geometry of the surface mating with the gasket (i.e., the flange surface), consisting of rings with sharp edges, promoted the formation of concentric contact areas, causing indentation of the flanges into the surface of the gaskets manufactured from photopolymer resin. Due to the relatively low mechanical strength of this material, the microstructure was already deformed under relatively low clamping forces of the flange joint. Additionally, with lower clamping forces, measurements were limited by the upper detection threshold of the helium detector used. Future research will be extended to include measurements conducted on a test stand enabling experiments within a wider range of gasket loads and with an increased leakage detection range;
- The numerical model was preliminary and simplified. The ring in the microstructure of the structured gaskets, as well as the roughness and waviness of the flange surfaces and the roughness of the gasket surfaces, were not accounted for, and water was adopted as the medium instead of the actual gas (helium) used in the experiment. To more precisely model the flow phenomena associated with leakage through the gasket, it is necessary to improve the numerical model. In future research, the use of a more accurate numerical model that incorporates the real gas will be considered.

It should be emphasized that the photopolymer resin used in the study is not a material commonly employed in gasket manufacturing. The primary objective of the research was to assess the potential of structuring the external sealing surface, which may be implemented in the future in gaskets manufactured from standard materials such as polytetrafluoroethylene (PTFE) or alloy steel.

## 4. Conclusions

The numerical calculations and experimental studies made it possible to assess the influence of the geometric parameters of the microstructure of the external sealing surface on the sealing performance of the flange joint. Based on these results, the following conclusions were formulated:

1. The application of a microstructure of the external sealing surface inspired by the operating principle of the Tesla one-way valve leads to improved sealing performance compared to a quasi-smooth gasket;
2. CFD simulations demonstrated that the introduction of a microstructure on a sealing surface can induce local circulation of the fluid, resulting in increased flow resistance and reduced leakage. The numerical calculations indicated that the critical geometric parameters influencing leakage reduction through the generation of fluid circulation are the gap width between the microprotrusions, the packing density of the microprotrusions, and their height;
3. Experimental studies confirmed the higher effectiveness of structured gaskets compared to quasi-smooth gaskets, particularly in the range of lower contact pressures;
4. An analysis accounting for the effective contact surface indicates that the improvement in sealing performance results both from intensified local contact pressures and from the flow effects generated by the microprotrusions;
5. In agreement with the predictions of the CFD calculations, the experimental results showed that among the structured gaskets, the highest sealing performance at the lowest contact pressures was achieved by Variant G2. The minimum leakage rate was 2.06 · 10^−4^ mg/(s·m) at a residual clamping force of 102.5 kN.

The obtained results confirm the potential for further development of the concept of external surface structuring. Future work should include experimental investigations in a wider range of gasket loads, combined with an increased leakage detection range, to better capture sealing performance under varied operating conditions. Further refinement of the numerical model is also planned, in particular by incorporating real gases (helium instead of water) and accounting for the full geometry of the microstructure (with the ring section), so as to more precisely represent the flow phenomena associated with gasket leakage. In addition, future studies will involve the use of other gasket materials, such as metals (e.g., aluminum, copper, or steel alloys) and manufacturing technologies (e.g., milling and laser texturing), in order to eliminate or reduce the influence of material properties on the achieved sealing performance.

## Figures and Tables

**Table 1:** Characteristic dimensions of the microstructure of the external sealing surfaces for all analyzed variants of structured gaskets.

Characteristic Dimension	G1	G2	G3	G4	G5	G6
Microstructure depth d, mm	0.1	0.1	0.1	0.4	0.1	0.1
Minor axis of ellipse $h_{e}$, mm	1.6	1.6	1.6	1.6	1.6	3.2
Height of the split of ellipse $h_{c}$, mm	0.08	0.08	0	0.08	0.08	0.16
Thickness of microprotrusions t, mm	0.3	0.3	0.3	0.3	0.15	0.3
Gap width $w_{g}$, mm	0.6	0.45	0.6	0.6	0.45	0.6
Number of microprotrusions in the circumferential direction $n_{circ}$	44	44	44	44	44	22
Number of microprotrusions in the radial direction $n_{rad}$	8	8	8	8	8	4

**Table 2:** Main parameters of calculation meshes.

Gasket Variant	Elements (Segment)	Elements (Full Geometry)	Average Orthogonal Quality	Average Skewness	Average Aspect Ratio
G0*	35,294,278	3,105,896,464	1.00000	0.000157	3.5064
G1	28,766,516	2,531,453,408	0.98596	0.043230	3.5573
G2	28,614,974	2,518,117,712	0.98617	0.041943	3.5602
G3	22,584,124	1,987,402,912	0.98941	0.035120	3.5151
G4	77,160,800	6,790,150,400	0.97051	0.073682	2.3141
G5	30,232,240	2,660,437,120	0.98696	0.041728	3.5407
G6	58,938,288	2,593,284,672	0.99284	0.026865	3.5359

**Table 3:** Mechanical properties of Formlabs Black V5 [45].

Property	Post-Cured at Room Temperature for 5 min	Post-Cured at 60 °C for 15 min
Ultimate Tensile Strength, MPa	57	61
Tensile Modulus, MPa	2450	2700
Elongation at break, %	14	10
Heat Deflection Temperature at 0.45 MPa, °C	61	69

**Table 4:** Specifications of the measurement instruments.

Measuring Instrument	Measurement Range	Accuracy
Helium pressure gauge RP T 95 62 (KFM, Wloclawek, Poland)	0–10 MPa	±0.05 MPa (CL 0.6)
Force sensor CL20-18 (ZEPWN, Marki, Poland)	0–200 kN	±0.01 kN
Helium leak detector LDS3000 (INFICON, Koln, Germany)	10^−7^–10^−1^ mbar·L/s	± 0.1 · 10^n^ mbar·L/s

**Table 5:** Images of selected tested samples.

Gasket Variant	Pre-Test Image	Post-Test Image
G3	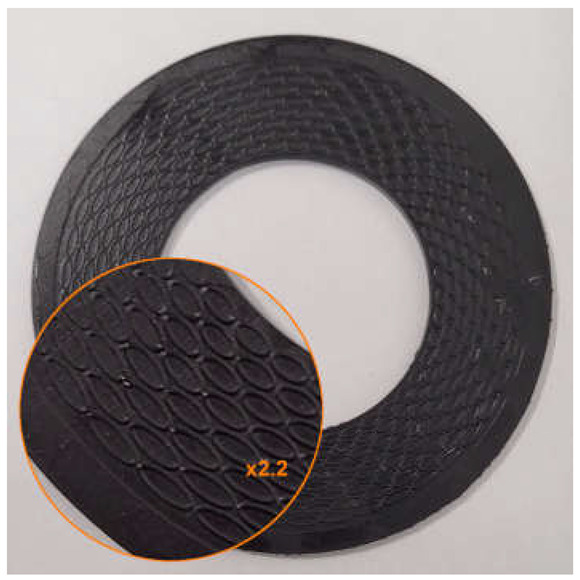	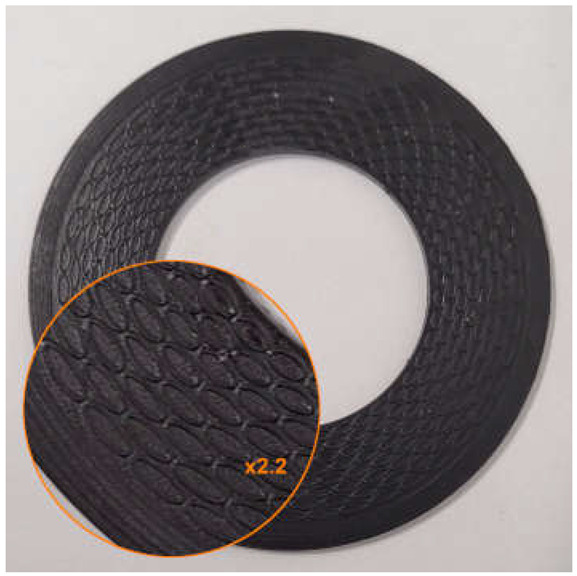
G4	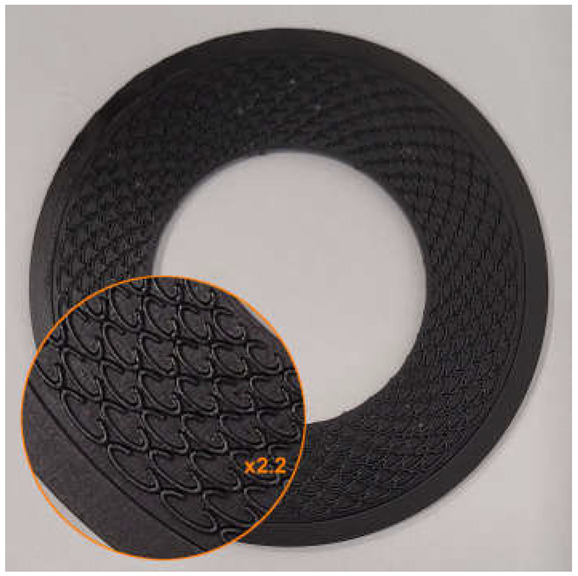	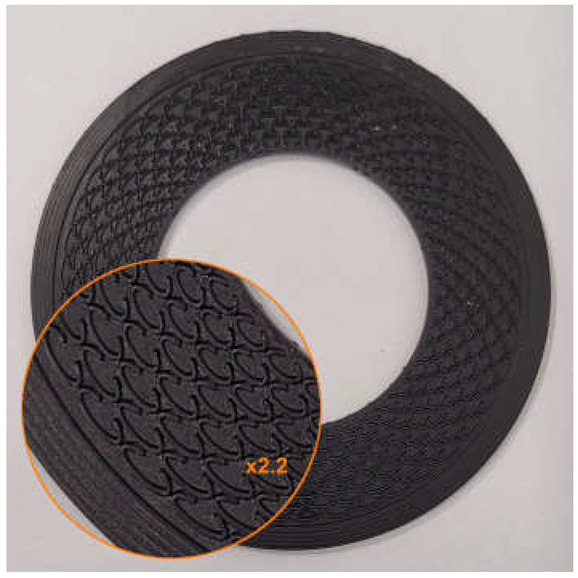
G6	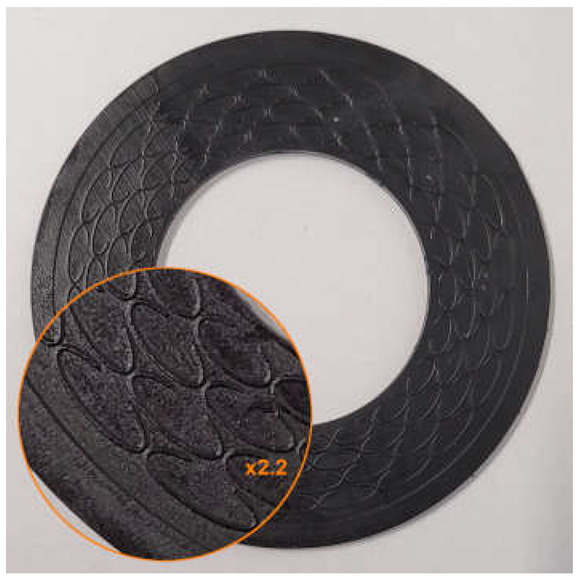	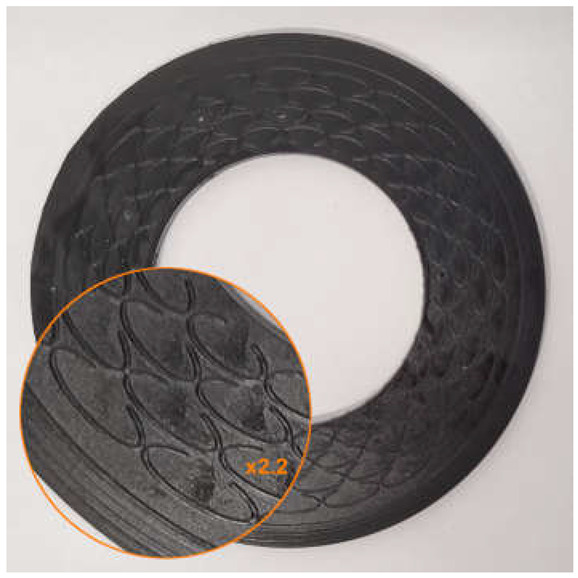

## Data Availability

The data presented in this study are available from the corresponding author upon reasonable request.

## Nomenclature

Symbol	Name	Unit
CFL	Courant-Friedrichs-Lewy number	-
d	microstructure depth (microprotrusions height)	mm
FRF	flow reduction factor	-
$h_{c}$	height of split of ellipse	mm
$h_{e}$	minor axis of ellipse	mm
m	mass	g
M	molar mass	g/mol
$n_{circ}$	number of microprotrusions in circumferential direction	-
$n_{rad}$	number of microprotrusions in radial direction	-
p	pressure	Pa
$q_{m}$	mass leakage rate	g/s
$q_{v}$	volumetric leakage rate	mbar·L/s
R	gas constant	J/(mol·K)
Re	Reynolds number	-
$R_{a}$	arithmetical mean height of profile	μm
$R_{z}$	maximum height of profile	μm
T	temperature	K
t	thickness of the microprotrusions	mm
V	volume	m^3^
$w_{g}$	gap width	mm
y+	value of y+ parameter	-
Subscripts		
n	index of gasket variant	-
